# 4-(1,2,4-Triazol-1-yl)aniline

**DOI:** 10.1107/S1600536810052025

**Published:** 2010-12-18

**Authors:** Hoong-Kun Fun, Ching Kheng Quah, B. Chandrakantha, Arun M. Isloor, Prakash Shetty

**Affiliations:** a X-ray Crystallography Unit, School of Physics, Universiti Sains Malaysia, 11800 USM, Penang, Malaysia; b Department of Chemistry, Manipal Institute of Technology, Manipal 576 104, India; c Organic Chemistry Division, Department of Chemistry, National Institute of Technology-Karnataka, Surathkal, Mangalore 575 025, India; d Department of Printing, Manipal Institute of Technology, Manipal 576 104, India

## Abstract

In the title compound, C_8_H_8_N_4_, the dihedral angle between the triazole ring [maximum deviation = 0.003 (1) Å] and the benzene ring is 34.57 (7)°. In the crystal, mol­ecules are linked into sheets lying parallel to the *ac* plane *via* inter­molecular N—H⋯N and C—H⋯N hydrogen bonds. Aromatic π–π [centroid–centroid distance = 3.6750 (8) Å] stacking and N—H⋯π inter­actions are also observed.

## Related literature

For general background to and the biological activity of triazole derivatives, see: Isloor *et al.* (2000 ▶, 2009 ▶); Soliman *et al.* (2001 ▶); Holla *et al.* (2000 ▶); Sunil *et al.* (2009 ▶). For bond-length data, see: Allen *et al.* (1987 ▶). For a related structure, see: Fun *et al.* (2010 ▶).
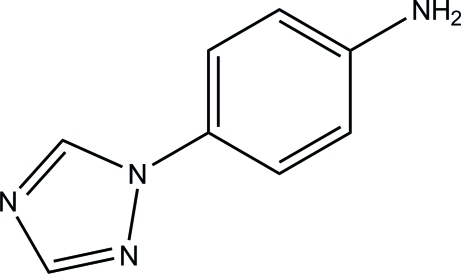


## Experimental

### 

#### Crystal data

C_8_H_8_N_4_
*M* *_r_* = 160.18
Monoclinic, 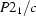

*a* = 5.5488 (1) Å
*b* = 7.3656 (2) Å
*c* = 19.5477 (5) Å
β = 99.416 (2)°
*V* = 788.15 (3) Å^3^
*Z* = 4
Mo *K*α radiation
μ = 0.09 mm^−1^
*T* = 296 K
0.50 × 0.42 × 0.14 mm

#### Data collection

Bruker SMART APEXII CCD diffractometer
Absorption correction: multi-scan (*SADABS*; Bruker, 2009 ▶) *T*_min_ = 0.957, *T*_max_ = 0.988
8036 measured reflections
2160 independent reflections
1722 reflections with *I* > 2σ(*I*)
*R*_int_ = 0.030

#### Refinement

*R*[*F* ^2^ > 2σ(*F* ^2^)] = 0.044
*wR*(*F* ^2^) = 0.118
*S* = 1.05
2160 reflections
110 parameters
H atoms treated by a mixture of independent and constrained refinement
Δρ_max_ = 0.23 e Å^−3^
Δρ_min_ = −0.17 e Å^−3^

### 

Data collection: *APEX2* (Bruker, 2009 ▶); cell refinement: *SAINT* (Bruker, 2009 ▶); data reduction: *SAINT*; program(s) used to solve structure: *SHELXTL* (Sheldrick, 2008 ▶); program(s) used to refine structure: *SHELXTL*; molecular graphics: *SHELXTL*; software used to prepare material for publication: *SHELXTL* and *PLATON* (Spek, 2009 ▶).

## Supplementary Material

Crystal structure: contains datablocks global, I. DOI: 10.1107/S1600536810052025/hb5764sup1.cif

Structure factors: contains datablocks I. DOI: 10.1107/S1600536810052025/hb5764Isup2.hkl

Additional supplementary materials:  crystallographic information; 3D view; checkCIF report

## Figures and Tables

**Table 1:** Hydrogen-bond geometry (Å, °) *Cg*2 is the centroid of the C3–C8 phenyl ring.

*D*—H⋯*A*	*D*—H	H⋯*A*	*D*⋯*A*	*D*—H⋯*A*
N4—H2*N*4⋯N1^i^	0.871 (16)	2.208 (16)	3.0709 (18)	171.1 (15)
C1—H1*A*⋯N2^ii^	0.93	2.50	3.4035 (16)	166
N4—H1*N*4⋯*Cg*2^iii^	0.87 (2)	2.58 (2)	3.3929 (16)	156.0 (17)

Symmetry codes: (i) 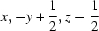; (ii) 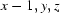; (iii) 
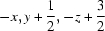
.

**Fig. 1.**
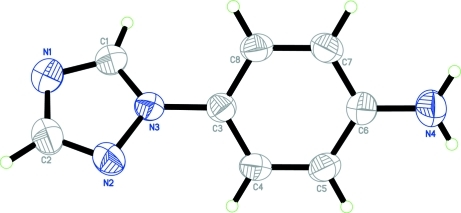
The molecular structure of the title compound showing 50% probability displacement ellipsoids for non-H atoms.

**Fig. 2.**
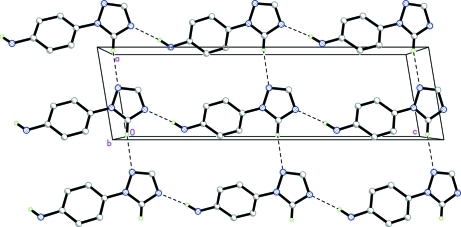
The crystal structure of the title compound, viewed along the b axis. H atoms not involved in hydrogen bonds (dashed lines) have been omitted for clarity.

## supplementary crystallographic information

### Comment

Compounds incorporating heterocyclic ring systems continue to attract considerable interests due to the wide range of biological activities they possess (Isloor *et al.*, 2000). Triazoles are a class of heterocyclic compounds having a five-membered ring of two carbon atoms and three nitrogen atoms (Soliman *et al.*, 2001). They have wide range of applications. In last few decades, triazoles have received much significant attention in the field of medicinal chemistry because of their diversified biological properties like antibacterial (Isloor *et al.*, 2009) and antifungal (Holla *et al.*, 2000) activities. In recent years, 1,2,4-triazole derivatives have been found to associate with anticancer properties (Sunil *et al.*, 2009). It is also observed that incorporation of aryl constituent into the triazoles ring systems augments the biological activities considerably.

In the title molecule (Fig. 1), the triazol-1-yl ring (N1-N3/C1/C2, maximum deviation = 0.003 (1) Å at atom N3) is inclined at angle of 34.57 (7)° with phenyl (C3-C8) ring. Bond lengths (Allen *et al.*, 1987) and angles are within normal ranges and are comparable to a related structure (Fun *et al.*, 2010).

In the crystal packing (Fig. 2), the molecules are linked into two-dimensional sheets parallel to the *ac*-plane *via* intermolecular N4–H2N4···N1 and C1–H1A···N2 hydrogen bonds. π-π stacking interactions between the centroids of N1-N3/C1/C2 triazol-1-yl rings (Cg1), with Cg1···Cg1^i^ distance of 3.6750 (8) Å [symmetry code: (i) 1-X, -Y, 2-Z] are observed. The crystal structure is further consilidated by N4–H1N4···Cg2 (Table 1) interactions, where Cg2 is the centroid of C3-C8 phenyl ring.

### Experimental

1,2,4-Triazole (2 g, 0.02 mol) was added lot-wise to a suspension of sodium hydride (60%, 1.47 g, 0.0308 mol) in dry DMF (20 ml) at 0°C. After the addition, the reaction mixture was stirred at the same temperature for 30 min. A solution of 4-fluoro nitrobenzene (2.82 g, 0.02 mol) in dry DMF (20 ml) was then added and the reaction mixture was stirred at room temperature for 18 h. The reaction mixture was then quenched with ice water and extracted with ethyl acetate. The organic layer was concentrated to afford a yellow solid as a nitro compound intermediate (3 g). This nitro compound was taken in methanol (30 ml) and hydrogenated using 10% palladium on carbon (0.2 g) at 3-kg pressure of hydrogen. After the reaction was over, the catalyst was filtered, the filtrate was concentrated to afford the title compound as a yellow solid.  Yellow blocks were recrystallised from ethanol. Yield : 2.8g, 60 %. *M.p.* 433-435K.

### Refinement

H1N4 and H2N4 were located in a difference Fourier map and allowed to refined freely. The remaining H atoms were positioned geometrically and refined using a riding model with C–H = 0.93 Å and *U*_iso_(H) = 1.2 *U*_eq_(C). The highest residual electron density peak is located at 0.67 Å from C3 and the deepest hole is located at 1.27 Å from C8.

### Crystal data

**Table:**

C_8_H_8_N_4_	*F*(000) = 336
*M**_r_* = 160.18	*D*_x_ = 1.350 Mg m^−^^3^
Monoclinic, *P*2_1_/*c*	Mo *K*α radiation, λ = 0.71073 Å
Hall symbol: -P 2ybc	Cell parameters from 3245 reflections
*a* = 5.5488 (1) Å	θ = 3.0–29.1°
*b* = 7.3656 (2) Å	µ = 0.09 mm^−^^1^
*c* = 19.5477 (5) Å	*T* = 296 K
β = 99.416 (2)°	Block, yellow
*V* = 788.15 (3) Å^3^	0.50 × 0.42 × 0.14 mm
*Z* = 4	

### Data collection

**Table:**

Bruker SMART APEXII CCD diffractometer	2160 independent reflections
Radiation source: fine-focus sealed tube	1722 reflections with *I* > 2σ(*I*)
graphite	*R*_int_ = 0.030
φ and ω scans	θ_max_ = 29.4°, θ_min_ = 2.1°
Absorption correction: multi-scan (*SADABS*; Bruker, 2009)	*h* = −7→7
*T*_min_ = 0.957, *T*_max_ = 0.988	*k* = −10→10
8036 measured reflections	*l* = −26→24

### Refinement

**Table:**

Refinement on *F*^2^	Secondary atom site location: difference Fourier map
Least-squares matrix: full	Hydrogen site location: inferred from neighbouring sites
*R*[*F*^2^ > 2σ(*F*^2^)] = 0.044	H atoms treated by a mixture of independent and constrained refinement
*wR*(*F*^2^) = 0.118	*w* = 1/[σ^2^(*F*_o_^2^) + (0.0509*P*)^2^ + 0.1531*P*] where *P* = (*F*_o_^2^ + 2*F*_c_^2^)/3
*S* = 1.05	(Δ/σ)_max_ = 0.001
2160 reflections	Δρ_max_ = 0.23 e Å^−^^3^
110 parameters	Δρ_min_ = −0.17 e Å^−^^3^
0 restraints	Extinction correction: *SHELXTL* (Sheldrick, 2008), Fc^*^=kFc[1+0.001xFc^2^λ^3^/sin(2θ)]^-1/4^
Primary atom site location: structure-invariant direct methods	Extinction coefficient: 0.042 (5)

### Special details

**Table:**

Geometry. All esds (except the esd in the dihedral angle between two l.s. planes) are estimated using the full covariance matrix. The cell esds are taken into account individually in the estimation of esds in distances, angles and torsion angles; correlations between esds in cell parameters are only used when they are defined by crystal symmetry. An approximate (isotropic) treatment of cell esds is used for estimating esds involving l.s. planes.
Refinement. Refinement of F^2^ against ALL reflections. The weighted R-factor wR and goodness of fit S are based on F^2^, conventional R-factors R are based on F, with F set to zero for negative F^2^. The threshold expression of F^2^ > 2sigma(F^2^) is used only for calculating R-factors(gt) etc. and is not relevant to the choice of reflections for refinement. R-factors based on F^2^ are statistically about twice as large as those based on F, and R- factors based on ALL data will be even larger.

### Fractional atomic coordinates and isotropic or equivalent isotropic displacement parameters (Å^2^)

**Table:**

	*x*	*y*	*z*	*U*_iso_*/*U*_eq_	
N1	0.3298 (2)	0.17124 (18)	1.08332 (6)	0.0542 (3)	
N2	0.58582 (19)	0.24895 (17)	1.00992 (6)	0.0498 (3)	
N3	0.34993 (17)	0.26004 (14)	0.97774 (5)	0.0378 (3)	
N4	0.0895 (3)	0.4820 (2)	0.70184 (6)	0.0597 (4)	
C1	0.2029 (2)	0.2126 (2)	1.02227 (7)	0.0480 (3)	
H1A	0.0334	0.2092	1.0117	0.058*	
C2	0.5611 (3)	0.1960 (2)	1.07255 (7)	0.0533 (4)	
H2A	0.6948	0.1768	1.1072	0.064*	
C3	0.2865 (2)	0.31773 (16)	0.90734 (6)	0.0360 (3)	
C4	0.4336 (2)	0.27300 (17)	0.85909 (6)	0.0411 (3)	
H4A	0.5752	0.2056	0.8724	0.049*	
C5	0.3691 (2)	0.32893 (18)	0.79107 (6)	0.0432 (3)	
H5A	0.4686	0.2986	0.7589	0.052*	
C6	0.1573 (2)	0.43007 (17)	0.76988 (6)	0.0404 (3)	
C7	0.0123 (2)	0.47403 (17)	0.81966 (6)	0.0434 (3)	
H7A	−0.1296	0.5415	0.8068	0.052*	
C8	0.0763 (2)	0.41891 (17)	0.88743 (6)	0.0415 (3)	
H8A	−0.0218	0.4496	0.9200	0.050*	
H2N4	0.171 (3)	0.447 (2)	0.6697 (8)	0.065 (5)*	
H1N4	−0.029 (3)	0.560 (3)	0.6921 (9)	0.078 (6)*	

### Atomic displacement parameters (Å^2^)

**Table:**

	*U*^11^	*U*^22^	*U*^33^	*U*^12^	*U*^13^	*U*^23^
N1	0.0549 (7)	0.0658 (8)	0.0435 (6)	0.0049 (6)	0.0124 (5)	0.0063 (5)
N2	0.0340 (5)	0.0681 (8)	0.0452 (6)	−0.0015 (5)	0.0006 (4)	0.0051 (5)
N3	0.0318 (5)	0.0448 (5)	0.0367 (5)	0.0009 (4)	0.0052 (4)	−0.0009 (4)
N4	0.0711 (8)	0.0683 (9)	0.0406 (6)	0.0212 (7)	0.0116 (6)	0.0085 (6)
C1	0.0392 (6)	0.0616 (8)	0.0447 (7)	0.0024 (6)	0.0115 (5)	0.0042 (6)
C2	0.0485 (7)	0.0650 (9)	0.0439 (7)	0.0014 (6)	−0.0002 (6)	0.0047 (6)
C3	0.0324 (5)	0.0396 (6)	0.0355 (6)	−0.0019 (4)	0.0043 (4)	−0.0023 (4)
C4	0.0313 (5)	0.0480 (7)	0.0445 (7)	0.0041 (5)	0.0074 (5)	−0.0005 (5)
C5	0.0388 (6)	0.0518 (7)	0.0412 (6)	0.0008 (5)	0.0133 (5)	−0.0019 (5)
C6	0.0427 (6)	0.0395 (6)	0.0386 (6)	−0.0027 (5)	0.0051 (5)	−0.0006 (5)
C7	0.0388 (6)	0.0453 (7)	0.0451 (7)	0.0081 (5)	0.0042 (5)	−0.0005 (5)
C8	0.0375 (6)	0.0468 (7)	0.0413 (6)	0.0053 (5)	0.0096 (5)	−0.0049 (5)

### Geometric parameters (Å, °)

**Table:**

N1—C1	1.3183 (17)	C3—C4	1.3842 (16)
N1—C2	1.3468 (19)	C3—C8	1.3851 (16)
N2—C2	1.3134 (18)	C4—C5	1.3821 (17)
N2—N3	1.3587 (14)	C4—H4A	0.9300
N3—C1	1.3332 (16)	C5—C6	1.3957 (17)
N3—C3	1.4284 (14)	C5—H5A	0.9300
N4—C6	1.3758 (16)	C6—C7	1.3987 (17)
N4—H2N4	0.871 (18)	C7—C8	1.3755 (16)
N4—H1N4	0.87 (2)	C7—H7A	0.9300
C1—H1A	0.9300	C8—H8A	0.9300
C2—H2A	0.9300		
			
C1—N1—C2	102.04 (11)	C8—C3—N3	119.62 (10)
C2—N2—N3	102.11 (11)	C5—C4—C3	119.72 (11)
C1—N3—N2	109.16 (10)	C5—C4—H4A	120.1
C1—N3—C3	128.78 (10)	C3—C4—H4A	120.1
N2—N3—C3	122.06 (10)	C4—C5—C6	121.17 (11)
C6—N4—H2N4	121.5 (11)	C4—C5—H5A	119.4
C6—N4—H1N4	118.2 (12)	C6—C5—H5A	119.4
H2N4—N4—H1N4	120.1 (16)	N4—C6—C5	121.27 (12)
N1—C1—N3	111.01 (12)	N4—C6—C7	120.72 (12)
N1—C1—H1A	124.5	C5—C6—C7	118.00 (11)
N3—C1—H1A	124.5	C8—C7—C6	120.95 (11)
N2—C2—N1	115.69 (12)	C8—C7—H7A	119.5
N2—C2—H2A	122.2	C6—C7—H7A	119.5
N1—C2—H2A	122.2	C7—C8—C3	120.16 (11)
C4—C3—C8	120.00 (11)	C7—C8—H8A	119.9
C4—C3—N3	120.38 (10)	C3—C8—H8A	119.9
			
C2—N2—N3—C1	−0.42 (15)	C8—C3—C4—C5	−0.32 (19)
C2—N2—N3—C3	178.65 (11)	N3—C3—C4—C5	179.64 (11)
C2—N1—C1—N3	−0.25 (16)	C3—C4—C5—C6	0.0 (2)
N2—N3—C1—N1	0.44 (16)	C4—C5—C6—N4	−178.34 (13)
C3—N3—C1—N1	−178.55 (12)	C4—C5—C6—C7	0.26 (19)
N3—N2—C2—N1	0.29 (17)	N4—C6—C7—C8	178.50 (13)
C1—N1—C2—N2	−0.04 (18)	C5—C6—C7—C8	−0.11 (19)
C1—N3—C3—C4	−146.03 (13)	C6—C7—C8—C3	−0.3 (2)
N2—N3—C3—C4	35.09 (17)	C4—C3—C8—C7	0.48 (19)
C1—N3—C3—C8	33.93 (19)	N3—C3—C8—C7	−179.49 (11)
N2—N3—C3—C8	−144.95 (12)		

### Hydrogen-bond geometry (Å, °)

**Table:**

Cg2 is the centroid of the C3–C8 phenyl ring.

**Table:**

*D*—H···*A*	*D*—H	H···*A*	*D*···*A*	*D*—H···*A*
N4—H2N4···N1^i^	0.871 (16)	2.208 (16)	3.0709 (18)	171.1 (15)
C1—H1A···N2^ii^	0.93	2.50	3.4035 (16)	166
N4—H1N4···Cg2^iii^	0.87 (2)	2.58 (2)	3.3929 (16)	156.0 (17)

Symmetry codes: (i) *x*, −*y*+1/2, *z*−1/2; (ii) *x*−1, *y*, *z*; (iii) −*x*, *y*+1/2, −*z*+3/2.
